# Laparoscopic Versus Robotic Yancey–Soave Primary Pull-Through in Rectosigmoid Hirschsprung Disease: A Systematic Review of the Literature

**DOI:** 10.3390/children13070846

**Published:** 2026-06-23

**Authors:** Lea A. Wehrli, Federico G. Seifarth

**Affiliations:** Pediatric Surgery, WVU Medicine Golisano Children’s Hospital, Morgantown, WV 26505, USA

**Keywords:** Hirschsprung disease, laparoscopy, minimally invasive surgery, laparoscopic-assisted pull-through, laparoscopic-assisted endorectal pull-through, robotic-assisted

## Abstract

**Objective:** Minimally invasive surgery in Hirschsprung disease (HSCR) management was introduced in the mid-1990s. Despite decades of clinical application of various laparoscopic approaches, there remains a paucity of high-powered prospective studies and comprehensive systematic reviews in the literature. This study aimed to systematically review and summarize published techniques and outcomes of laparoscopic- and robotic-assisted surgery in HSCR. **Methods:** A systematic literature review was conducted using PubMed and the Cochrane Library. Studies reporting technical and outcome data of laparoscopic- or robotic-assisted surgery for HSCR were included. Data extraction and analysis were performed in accordance with the PRISMA 2020 guidelines. Parameters of interest included surgical technique, age at primary pull-through (PT), operative time, and functional outcomes. Outcomes of laparoscopic- versus robotic-assisted Yancey–Soave PT were compared. **Results:** A total of 700 publications were screened, of which seven studies met the inclusion criteria. Data from 556 patients were analyzed. A total of 338 underwent laparoscopic-assisted, and 218 underwent robotic-assisted pull-through. Large variability of the reported transanal resection technique (modified Yancey–Soave PT) was reported. Four studies reported functional outcomes in patients aged over four years. Three studies directly compared laparoscopic- and robotic-assisted PT; two reported no difference in the incidence of postoperative Hirschsprung-associated enterocolitis (HAEC). Functional outcomes were assessed using the Krickenbeck classification in three studies and the bowel function score in one study, with no significant differences reported in patients aged >4 years. **Conclusions:** Laparoscopic- and robotic-assisted Yancey–Soave PT appears to be safe for HSCR. Large variability in the applied surgical technique—despite being commonly classified as modified Yancey–Soave PT—as well as heterogeneity in the bowel function assessment, limit direct comparability between studies. To date, no single minimally invasive approach has demonstrated clear superiority over others. Prospective, randomized controlled studies are required to enable robust comparative evaluation of techniques, overall costs, and outcomes.

## 1. Introduction

Hirschsprung disease (HSCR) results from failed migration of neural crest cells during embryonic development, leading to an absence of enteric ganglion cells in variable lengths of the distal intestine [1,2], with an estimated incidence of 1 in 5000 live births [3]. The resulting aganglionic segment causes functional obstruction, most commonly confined to the rectosigmoid colon (75–80% of cases). Historically, management involved a three-stage approach: initial biopsies with leveling ostomy, laparotomy with definitive pull-through (PT), and ostomy closure.

Several minimally invasive approaches have been described in treating HSCR, including single-incision laparoscopic surgery (SILS), conventional multiport laparoscopic-assisted or robotic-assisted surgery, each combined with either a transanal Swenson or Yancey–Soave PT procedure. For the purposes of this review, the procedure is designated as the Yancey–Soave PT. Although this terminology was formally introduced in 2022–2024, all studies published prior to this period describe the surgical technique under the terms Soave or modified Soave PT [4,5]. These studies are therefore interpreted as reporting outcomes of the Yancey–Soave procedure.

This review provides a critical overview of minimally invasive techniques for non-syndromic HSCR involving the rectosigmoid and descending colon, highlighting clinical and functional outcomes of LAS and RAS transanal PT procedures. Despite numerous studies, significant heterogeneity and a lack of direct comparative data persist, underscoring an important knowledge gap in the optimal surgical approach for HSCR.

## 2. Materials and Methods

A comprehensive literature search was conducted using the PubMed database and Cochrane Library for studies published between 1992 and 2026. The literature search strategy incorporated Medical Subject Headings (MeSH) combined with Boolean operators (“AND,” “OR”). The primary search string was constructed as follows: (“Hirschsprung”) [MeSH] AND (“minimally invasive” or “laparoscopy” OR “laparoscopic” OR “robotic”).

This approach aimed to identify studies evaluating minimally invasive surgical techniques, including both laparoscopic- and robotic-assisted procedures in rectosigmoid and descending colonic HSCR. Inclusion criteria were: articles describing laparoscopic- or robotic-assisted Yancey–Soave or modified Yancey–Soave PT for HSCR, HSCR extending at most to the descending colon, systematic reviews, review articles, randomized controlled trials, non-randomized comparative studies (controlled clinical trials and cohort studies), and case series with >2 patients. Exclusion criteria were: open surgical techniques for HSCR treatment, previous colostomy, HSCR extending past the distal colon, total colonic or syndromic HSCR, multi-stage, redo or revisional surgery, letter/comments on articles, case reports, meta-analyses, conference abstracts or if the abstract was only available, articles not published or available in English or if the patient cohort was ≥18 years of age. Studies that did not report postoperative outcomes were also excluded from further analysis.

Retrieved records were deduplicated prior to screening. Study selection was conducted in two sequential phases. First, a literature search was conducted, then all abstracts were screened and studies that did not meet the predefined inclusion criteria were excluded. In the second phase, full-text articles were obtained and systematically assessed for eligibility, based on the established criteria. Additionally, the reference list of retrieved articles was screened for any possible further studies. A structured citation table was subsequently developed to catalog the included studies. The included studies were independently evaluated and reviewed by the two authors. Assessment of risk bias was conducted using validated tools for each included study type, either Joanna Briggs Institute Critical Appraisal Checklist for Case Series, the Newcastle–Ottawa Scale for non-randomized comparative cohort studies, or the Risk of Bias 2 assessment for randomized controlled studies [6,7,8]. Any discrepancies in assessment were resolved through discussion and consensus.

## 3. Results

**Figure 1:**
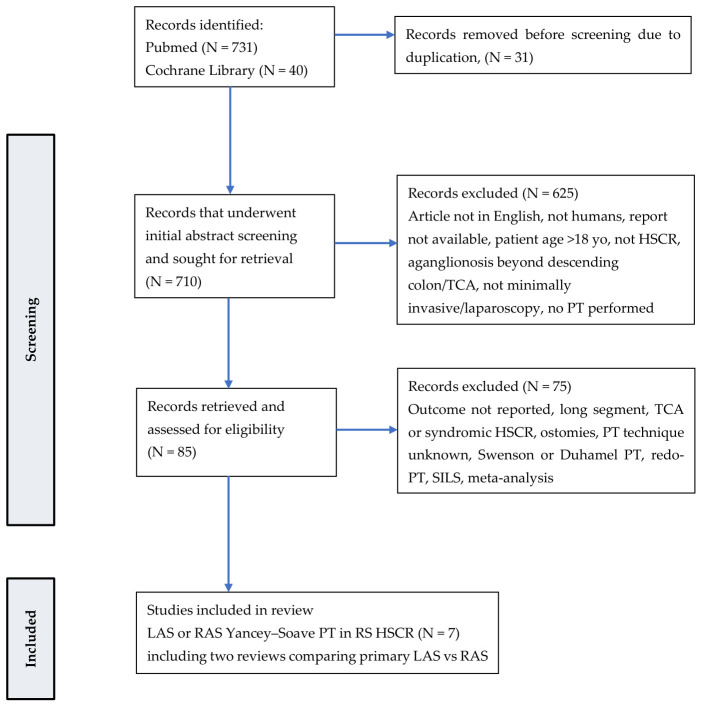
PRISMA 2020 flow diagram showing screening process for initial search results including: “HSCR” and “Minimally invasive” or “Laparoscopy” or “Laparoscopic” as well as “HSCR” and “Robotic”. Abbreviations: TCA = Total colonic aganglionosis. PT = Pull-through. RS HSCR = Rectosigmoid Hirschsprung disease. SILS = Single-incision laparoscopic surgery. LAS = Laparoscopic-assisted surgery. RAS = Robotic-assisted surgery.

A total of 741 articles were identified describing the therapeutic application of minimally invasive surgery, including 703 reports of laparoscopic techniques and 38 publications for robotic approaches in HSCR (Figure 1). The most frequently reported surgical techniques used were a Yancey–Soave PT or modified Yancey–Soave PT.

A total of 692 studies were excluded. To increase homogeneity of the included literature, studies were further excluded if they reported Duhamel or Swenson PT, single-incision laparoscopy, or if the surgical technique was not described. Studies reporting outcomes of minimally invasive Swenson pull-through procedures included heterogeneous patient populations, encompassing various lengths of aganglionic segments, patients with preexisting colostomies or comorbidities, and modifications to the original Swenson technique. Therefore, these studies were excluded from the present review.

Seven studies met inclusion criteria, of which three compared outcomes between laparoscopic- and robotic-assisted transanal modified Yancey–Soave PT [9,10,11,12,13,14,15]. One study was a prospective multicenter study, while the remaining six were retrospective single-center studies. Studies reporting laparoscopy were published from 2015 to 2025 while studies reporting robotic-assisted PT were from 2022 to 2025. Of the 546 patients, a total of 338 patients underwent laparoscopic-assisted transanal modified Yancey–Soave PT (Table 1 and Table 2). Additionally, nine meta-analyses were identified (Table 3).

Risk of bias assessment was conducted using study-design-appropriate, validated tools for each included study type, with results summarized in Table 4.

While assessing the reported surgical approaches, considerable variability was observed in the technique described as a modified Yancey–Soave PT. The minimal distance to the dentate line for the incision of transanal endorectal approach was reported to be 3 mm and up to 2 cm in older children [15,24]. One study reported splitting the muscular cuff both anteriorly and posteriorly [11], others promoted not splitting it at all [12,24,25], resecting the posterior muscular cuff completely [13], or creating a v-shaped muscular cuff [14,26,27]. Variable length of the muscular cuff, with some describing a longer cuff anteriorly than posteriorly, are also described [15,28,29]. Variations in the creation of the coloanal anastomosis have been reported, including heart-shaped [30] and oblique configurations [31]. In the robotic approach, dissection has been described in multiple planes, including perirectal [32], subserosal [14], seromuscular [33], and endorectal [34]. As described by some authors, the benefits of the robotic approach would allow further distal dissection almost to the anal canal, requiring only a very short transanal endorectal dissection of 0.3–0.5 cm [14].

The three studies comparing LAS to RAS reported no significant differences in age and weight at the time of surgery, gender ratio, and level of aganglionosis (Table 5 and Table 6) [12,13,14]. Length of surgery varied significantly between studies. Zhang et al. reported significantly shorter operative time with longer transanal dissection in the LAS group. Li Y found that operative time with RAS as well as transanal dissection duration was significantly shorter [13,14]. Estimated intraoperative blood loss was reported in three studies. Two studies demonstrated significantly lower blood loss with robotic-assisted surgery, whereas one study found no statistically significant difference between laparoscopic- and robotic-assisted Yancey–Soave pull-through procedures [12,13,14].

Anastomotic complications, strictures, and Hirschsprung-associated enterocolitis (HAEC) occurred significantly more frequently in the LAS group of Li Y’s study than in the RAS group. In contrast, Li W found no significant differences in occurrence of anastomotic complications or HAEC.

Interestingly, the study by Zhang was the only one who performed an anorectal manometry one year postoperatively. No significant differences were found in anal canal resting pressure or high-pressure zone length comparing LAS to RAS [14].

Functional outcomes were assessed with the Krickenbeck classification in three [10,11,15], bowel function score in one [14], and the postoperative fecal continence score (POFC) in two reports [13,14]. No significant differences within the seven items as well as the overall bowel function score was found in patients older than 4 years of age (LAS 17.09 ± 2.18 versus RAS 17.23 ± 2.25, *p* = 0.398) [14]. In the one-year POFC outcomes, no significant difference was found in defecation frequency as well as overall scores for patients older than two years of age. None suffered from fecal incontinence [13].

## 4. Discussion

Various minimally invasive approaches in HSCR have been described in the literature, encompassing both diagnostic and therapeutic applications. These techniques include minimally invasive colonic biopsy as well as laparoscopic- and robot-assisted pull-through (PT) procedures. Once the diagnosis of Hirschsprung disease has been confirmed by rectal biopsy, the surgical management is determined based on the length of the aganglionic segment. The primary PT procedure in patients with rectosigmoid HSCR entails a minimally invasive approach with laparoscopic-assisted colonic (rectosigmoid) biopsies followed by the PT procedure under the same anesthesia [35]. Patients with insufficient colonic decompression despite rectal irrigations, concerns for long-segment HSCR, signs of enterocolitis, or difficulties to access healthcare are poor candidates for primary PT procedure. If reliable intraoperative histopathological workup by frozen biopsies is not readily available, biopsies should be taken for definitive work up and a temporizing ileostomy should be created [36]. As suggested by Georgeson, it is essential to establish the level of the transition zone at the beginning of the intraabdominal procedure [37]. In his original work, seromuscular biopsies were obtained laparoscopically. This technique has since been further refined to laparoscopic-assisted full-thickness biopsies, performed either intracorporeally via trocars or extracorporeally through a limited umbilical incision, to accurately define the transition zone between aganglionic and ganglionated bowel [24,38].

The utility of laparoscopy in the surgical treatment of HSCR was first described in August 1994 by Smith. He reported a case of a laparoscopic Duhamel PT in a two-year-old boy. Curran and Raffensberger reported the successful outcomes and feasibility of laparoscopic Swenson PT in mongrel dogs in September 1994 [21,39]. This was shortly followed by Georgeson’s description of a primary laparoscopic-assisted endorectal Yancey–Soave PT in 12 patients in 1995 [40]. In Georgeson’s initial report, the muscular cuff was split posteriorly to a level of 3 cm proximal to the dentate line. Subsequent refinements have led to what is commonly termed the ‘modified Yancey–Soave pull-through’, involving an initial mucosectomy followed by a transition to full-thickness dissection in the proximal bowel. In 2010, Muensterer and Georgeson reported their experience with single-incision laparoscopic endorectal PT in six infants. As stated by the authors themselves, although technically challenging, it can be performed safely with good postoperative results and excellent cosmesis [41]. The following year, Hebra presented the first outcomes of twelve infants, who underwent robotic Swenson PT in 2011 [12]. Since these early reports, minimally invasive PT procedures for HSCR have been widely adopted, and are now considered the standard approach for short-segment Hirschsprung disease [42,43].

The standard reported laparoscopic approach for rectosigmoid and descending HSCR is a Yancey–Soave PT. In our literature search, only seven reports on either laparoscopic- or robotic-assisted Swenson PT were found, with the most recent being published by Hou in 2025 [31]. This surgical approach carries higher risks of injuring pelvic innervation during dissection, which is avoided by the Yancey–Soave approach due to its submucosal rather than full thickness rectal dissection. Laparoscopic applications of the Duhamel procedure, once very popular, have largely fallen out of favor for the treatment of rectosigmoid HSCR. The most recent systematic review and meta-analysis comparing open to laparoscopic Duhamel PT in non-TCA dates back to 2016 [44]. A recent study showed that a Duhamel PT is safer when compared to a Swenson PT in patients with TCA [45].

A recent meta-analysis by Durazo et al. including 291 patients showed significant lower blood loss in RAS compared to LAS, aligning with the results presented by Li Y and Zhang [13,14,46]. No significant difference was observed in operative times between the two groups, however significantly higher hospitalization expenses were reported in the RAS group with a mean difference of 44,922 CNY (6200 USD) compared to the LAS group [13]. This is consistent with the report by Hou, which indicated that robotic surgery costs approximately 25,000 CNY (3500 USD) more than laparoscopic surgery [31]. No studies to date have compared the costs of robotic- versus laparoscopic-assisted PT surgery in HSCR in the United States. The authors concluded that due to limited sample sizes, variability of the applied surgical approaches and study methodologies, cautious interpretation of the findings is recommended [46]. In the meta-analysis and review by Durazo, all four included studies originated from China, with two publications authored by the same research group. As noted in the 2025 meta-analysis, a serious risk of bias, as well as significant concerns regarding potential confounding factors, were identified [46].

In general, laparoscopic and robotic approaches in HSCR and their outcomes are well documented and have been established as safe procedures. Besides proper surgical techniques, the success of the procedure remains ultimately highly dependent on accurate intraoperative histopathological assessment. Confirmation of ganglionated bowel through biopsies is essential to guide the level of resection and ensure a PT of a well ganglionated segment. Additionally, preservation of the anal canal during the surgical procedure is of high importance, since damage to the anal canal can result in permanent fecal incontinence [47,48]. Comparative evaluation of techniques and outcomes between laparoscopic-assisted and robotic-assisted modified Yancey–Soave PT remains challenging. This is largely due to heterogeneity in surgical techniques with multiple modifications of the coloanal anastomosis, while still considered a modified Yancey–Soave PT [27,30].

Functional outcomes, particularly fecal continence, are frequently assessed using non-validated scoring systems. The absence of a standardized, validated pediatric-specific scoring system limits the reliability and comparability of reported outcomes across studies [49,50]. Furthermore, long-term outcome data remain limited. Although postoperative bowel function has been reported in adult patients, the distinction between patient subgroups and the assessment of their respective bowel functional outcomes remain challenging [51,52]. Although several authors reported no cases of fecal incontinence, fecal soiling was described in up to 61% of patients [10]. This discrepancy raises concerns that underlying fecal incontinence may not have been adequately assessed at the time of reporting. It further underscores the need for standardized postoperative assessment of bowel function and continence, as well as long-term follow-up.

This review has several limitations. Only one of the included studies was a prospective, randomized controlled trial with a follow-up of more than four years. The remaining studies were retrospective, with variable follow-up durations, and exhibited considerable heterogeneity in study populations, surgical techniques, and outcome measures. Several non-English publications were excluded, potentially limiting access to relevant data and introducing selection bias. Despite restricting the review to patients with rectosigmoid HSCR treated with laparoscopic- or robotic-assisted primary Yancey–Soave PT, considerable heterogeneity in outcome reporting persisted. Moreover, the inconsistent use of standardized, validated measures of bowel function and fecal continence limited cross-study comparability and hindered the synthesis of functional outcomes. The descriptive nature of the synthesis, while necessary due to these limitations, precludes formal quantitative conclusions.

## 5. Conclusions

Laparoscopic- and robot-assisted techniques are well established in the management of patients with Hirschsprung disease. These approaches are considered safe and offer the established benefits of minimally invasive surgery compared with open techniques. Large, multicenter randomized controlled trials with long-term follow-up extending well beyond four years of age are needed to evaluate the outcomes and potential advantages of minimally invasive surgery in patients with Hirschsprung disease. Furthermore, the use of standardized, validated instruments for the assessment of bowel function and fecal continence is encouraged to ensure reliable and comparable outcome reporting. In addition, economic evaluations assessing cost implications are warranted for comparativeness between surgical techniques.

## Figures and Tables

**Table 1:** Included studies reporting laparoscopic-assisted Yancey–Soave PT.

Author	Year	Country	Study Period	Study Design	Center	Extent of Aganglionosis	Surgery
Nam et al. [11]	2015	Korea	2010–2012	Retrospective	Single center	Rectosigmoid	Modified Yancey–Soave PT
Guerra et al. [9]	2016	Canada	1995–2014	Retrospective	Single center	Rectosigmoid	Yancey–Soave PT
Chan et al. [15]	2021	China	2002–2017	Retrospective	Single center	Rectosigmoid	Modified Yancey–Soave PT
Li W et al. [12]	2022	China	2015–2019	Retrospective Comparative	Single center	RS, desc	Modified Yancey–Soave PT
Wang et al. [10]	2025	China	2016–2021	Retrospective	Single center	Rectosigmoid	Yancey–Soave PT
Li Y et al. [13]	2025	China	2021–2023	Retrospective Comparative	Single center	Rectosigmoid	Modified Yancey–Soave PT
Zhang et al. [14]	2025	China	2015–2022	Prospective	Multicenter	RS, desc	Modified Yancey–Soave PT

Abbreviations: RS, desc: Aganglionosis within rectosigmoid and descending colon. PT = Pull-through.

**Table 2:** Included studies reporting robotic-assisted Soave PT.

Author	Year	Country	Study Period	Study Design	Center	Extent of Aganglionosis	Surgery
Li W et al. [12]	2022	China	2015–2019	Retrospective Comparative	Single center	RS, desc	Modified Yancey–Soave
Li Y et al. [13]	2025	China	2021–2023	Retrospective Comparative	Single center	Rectosigmoid	Modified Yancey–Soave
Zhang et al. [14]	2025	China	2015–2022	Prospective	Multicenter	RS, desc	Modified Yancey–Soave

Abbreviations: RS, desc: Aganglionosis within rectosigmoid and descending colon.

**Table 3:** Reported systematic reviews and meta-analyses in patients with HSCR from 2015 to 2026.

Meta-Analysis				
Approach	Author	Year	Studies Included	Analysis
Lap	Zhang et al. [16]	2015	16	Comparison Lap assisted vs. Laparotomy
Lap/transanal	Thomson et al. [17]	2015	5	Comparison Lap assisted vs. transanal endorectal PT
Lap	Tomuschat et al. [18]	2016	16	Comparison Lap assisted PT surgery types
Lap/transanal	Dai et al. [19]	2020	12	Outcome Quality of Life
Lap/transanal	Wang et al. [10]	2025	6	Comparison Lap assisted vs. transanal endorectal PT
Robo/Lap	Langeron et al. [20]	2025	16	Comparison Roboic vs. Laparoscopic-assisted
Robo/Lap	Durazo et al. [21]	2025	4	Outcome Robotic vs. Laparoscopic-assisted PT
Robo/Lap	Li Z et al. [22]	2025	6	Outcome Robotic vs. Laparoscopic-assisted PT
Robo	Rizvi et al. [23]	2026	6	Outcome Robotic-assisted Yancey–Soave PT

**Table 4:** Assessment of risk of bias.

Study	Design	Risk Assessment Tool	Overall Risk Assessment
Nam et al. [11]	Retrospective case series	JBI Critical Appraisal Checklist for Case Series	High risk of bias due to case series design. No comparative group.
Guerra et al. [9]	Retrospective matched comparative cohort and meta-analysis	NOS for Cohort Studies	Moderate risk of bias. 6 out of 9 stars. Timing of bowel function outcome not reported and not assessed using a standardized scoring system.
Chan et al. [15]	Single-arm retrospective cohort	JBI Critical Appraisal Checklist for Case Series	Moderate risk of bias. No comparative group. The 15-year enrollment period raises concerns for heterogeneity in management, surgical practices and care.
Li W et al. [12]	Three-arm retrospective comparative cohort	NOS for Cohort Studies	Moderate risk of bias. 5 out of 9 stars. Incomplete follow-up reporting and unclear adjustment for confounders.
Wang et al. [10]	Retrospective comparative cohort and systematic review/meta-analysis	NOS for Cohort Studies	Moderate risk of bias. 6 out of 9 stars. Non-random treatment allocation based on surgeon preference and time period. Potential recall bias due to parent-reported bowel function outcomes collected via telephone interviews.
Li Y et al. [13]	Two-arm retrospective comparative cohort	NOS for Cohort Studies	Moderate risk of bias. 6 out of 9 stars. Non-random treatment allocation based on surgeon preference and equipment availability. Incomplete follow-up reporting and unclear adjustment for confounders.
Zhang et al. [14]	Randomized controlled trial	RoB2 assessment	High risk of bias. 3 out of 5 domain. Unclear randomization despite concealed envelopes; missing bowel function data in approximately 1/3 of participants due to age-related limitations; retrospective trial registration.

Abbreviations: JBI: Joanna Briggs Institute, NOS: Newcastle–Ottawa Scale, RoB2: Risk of Bias 2.

**Table 5:** (**A**) Outcomes of included studies reporting laparoscopic-assisted Yancey–Soave PT. (**B**) Functional outcomes of included studies reporting laparoscopic-assisted Yancey–Soave PT.

**(A)**						
**Author**	**Cohort Male/Female**	**Age at Surgery [Months]**		**OR Time [min]**	**Blood Loss [mL]**	
Nam et al. [11]	8 5/3	118		263	No transfusion	
Guerra et al. [9]	24 16/8	3.5		234	No transfusion	
Chan et al. [15]	41 37/4	1.8		240	NA	
Li W et al. [12]	30 20/10	4.3		152	9.1 ± 2.2 mL	
Wang et al. [10]	37 31/6	19		NA	NA	
Li Y et al. [13]	35 25/10	32		203	16.9 ± 13.2 mL	
Zhang et al. [14]	163 113/50	8.6		117	10 (8–23) mL	
**(B)**						
**Author**	Soiling/BFS *	**Constipation**	**Enterocolitis**	**Anastomotic** **Leakage**	**Stricture**	**Mean F/U** **[Months]**
Nam et al. [11]	1(12.5%)	5 (62.5%)	0	0	0	37
Guerra et al. [9]	NA	0	3 (12.5%)	0	3 (12.5%)	NA
Chan et al. [15]	14 (34.1%)	0	7 (17%)	2 (4.9%)	0	108
Li W et al. [12]	2 (6.7%)	1 (3.3%)	5 (16.7%)	1 (3.3%)	0	36
Wang et al. [10]	16 (61.5%)	1 (3.8%)	4 (10.8%)	0	0	48.5
Li Y et al. [13]	3.8 ± 2.2 soiling/week	NA	5 (14.3%)	2 (5.7%)	3 (8.6%)	12
Zhang et al. [14]	BFS 17.09 ±2.18	NA	16 (9.82%)	4 (2.42%)	1 (0.61%)	>48

Abbreviations: NA = non-available. * Soiling/BFS: Soiling is reported as percentage or weekly frequency; bowel function score (BFS) values are presented as reported.

**Table 6:** (**A**) Outcomes of included studies reporting robotic-assisted Yancey–Soave PT. (**B**) Functional outcomes of included studies reporting laparoscopic-assisted Yancey–Soave PT.

**(A)**						
**Author**	**Cohort M/F**	**Age at Surgery [Months]**		**OR Time [min]**	**Blood Loss [mL]**	
Li W et al. [12]	28 18/10	4.3		180	10.2 ± 3.2 mL	
Li Y et al. [13]	25 17/8	37.8		180.9	11.0 ± 6.0 mL	
Zhang et al. [14]	165 111/54	9		156	5 (3–12) mL	
**(B)**						
**Author**	Soiling/BFS *	**Constipation**	**Enterocolitis**	**Anastomotic Leakage**	**Stricture**	**Mean F/U [Months]**
Li W et al. [12]	2 (7.1%)	1 (3.6%)	4 (14.3%)	1 (3.6%)	NA	36
Li Y et al. [13]	1.2 ± 0.5 soiling/week	NA	1 (4%)	0	0	12
Zhang et al. [14]	BFS 17.23 ± 2.25	NA	17 (10.3%)	4 (2.45%)	2 (1.21%)	>48

* Soiling/BFS: Soiling is reported as percentage or weekly frequency; bowel function score (BFS) values are presented as reported.

## Data Availability

No new data were created or analyzed in this study. Data sharing is not applicable to this article.
